# Information literacy of U.S. and Indian engineering undergraduates

**DOI:** 10.1186/2193-1801-2-244

**Published:** 2013-05-27

**Authors:** Roman Taraban, Damodar Suar, Kristin Oliver

**Affiliations:** Department of Psychology, Texas Tech University, Lubbock, TX USA; Department of Humanities & Social Sciences, Indian Institute of Technology Kharagpur, West Bengal, India; Department of Psychology, Texas Tech University, Lubbock, TX USA; Department of Educational Leadership, Texas Tech University, Lubbock, TX USA

**Keywords:** Cross-cultural differences, Information literacy, Text processing strategies, Epistemological beliefs

## Abstract

To be competitive, contemporary engineers must be capable of both processing and communicating information effectively. Available research suggests that Indian students would be disadvantaged in information literacy in their language of instruction (English) compared to U.S. students because English is not Indian students’ native language. Compared to U.S. students, Indian students (a) were predicted to apply practical text processing strategies to a greater extent than analytic strategies and (b) endorse the direct transmission of information over critical, interpretive analysis of information. Two validated scales measuring self-reported use of reading strategies and beliefs about interpreting and critiquing written information were administered to engineering students at an Indian Institute of Technology in their freshman to senior years. Neither prediction was supported: Indian students reported applying analytic strategies over pragmatic strategies and were more disposed to critically analyze information rather than accept it passively. Further, Indian students reported being more analytic and more reflective in their reading behaviors than U.S. engineering students. Additional data indicated that U.S. and Indian students’ text-processing strategies and beliefs are associated with the texts that they read and their academic behaviors.

**Electronic supplementary material:**

The online version of this article (doi:10.1186/2193-1801-2-244) contains supplementary material, which is available to authorized users.

## Background

The process of globalization has changed economies and the workplace worldwide, and with it, the parameters of training workplace-ready graduates. Language and communication play a major role. Indeed, on the question of employability, Farrell et al. (2005 found that language ability was a concern of human resource professionals when considering graduates from countries like India. Doubts about the quality of Indian graduates have appeared in the public media. The U.S. publication, *The Wall Street Journal* (Anand 2011), recently posted a headline that India graduates were not fit to hire. In part, “The average graduate’s ability to comprehend and converse is very low” (p. A12). A leading news magazine, *India Today* (2012), reported that “Twenty five to 35 per cent engineers cannot comprehend English usage even in day-to-day conversations. Since engineering education is in English, this is a key concern for colleges, as such a lack inhibits students from grasping concepts in other subject areas as well.” There is a strong perception in the media that Indian graduates lack the English language skills necessary for success in the workplace.

Assertions in the media about Indian students set the stage for the present research, but are not the primary motivation. Rather, this study is motivated by psycholinguistic research that shows that individuals process language differently in their native language compared to a non-native language (Vianty 2007). Indian students may indeed find English difficult and may process it differently because English is not their native language. This raises the question of whether U.S. graduates have a language advantage because English is their native language, not a second-language, compared to Indian engineering students for whom English is not a native language. The present research compares the ability of U.S. and Indian engineering undergraduates to process and evaluate written materials.

Preparing for the workplace is challenging. Engineers must learn not only the technical skills of their disciplines but also “the way engineers talk, write, think, and approach problems” (Bielenberg 2011 p. 2). A crucial element of academic training involves developing the ability for critical and creative thinking (Pavelich and Moore 1996). Educators stress the importance of processing information in a deep rather than in a superficial manner, which is termed *information literacy* in this paper. Information literacy refers to skills, attitudes, knowledge, and a range of ways of processing information (Starkey et al. 2006). Processing information is intricate and complex (Shuman et al. 2005), and requires an ability to think critically and creatively (MacAlpine and Uddin 2009). Processing of written information (a) is guided by a person’s beliefs about the role and purpose of written information, here referred to as *epistemological beliefs*, and (b) requires applying *metacognitive strategies* to guide the mental processes of comprehension and analysis. The constructs of epistemological beliefs and metacognitive strategies are used to compare U.S. and Indian engineering students in this paper.

### Epistemological beliefs

The information that a person gains from text depends in part on what the person thinks he or she should gain from the text. Research has shown that individuals hold different expectations about the knowledge they will gain (Mason et al. 2006;Schraw 2000). Some individuals regard the author as an authority and expect text to communicate reliable facts. Others regard reading as a constructive process in which readers take an active role in their comprehension of the material by questioning, evaluating, and responding to the author (Wyatt et al. 1993). Readers’ expectations regarding written sources of information and their responses to that information define their epistemological beliefs (Schraw 2000). A person will negotiate the text differently depending on those beliefs. Individuals’ epistemological beliefs determine whether they will actively process information in a critical and analytic manner or whether they will passively depend on the author to communicate information factually and reliably.

Epistemological beliefs assess two kinds of beliefs about the purpose of text: (a) *transmission* and (b) *transaction* (Schraw 2000). Transmission beliefs treat text as a means of direct communication between author and reader, without interpretation (e.g., an item from the transmission subscale: *The main purpose of reading is to understand what the author says*). If readers hold this view, they expect the author to communicate factual information in a direct fashion. The author is the authority. Transaction beliefs emphasize the construction of knowledge by individuals (e.g., an item from the transaction subscale: *I enjoy interpreting what I read in a personal way*). When readers adopt a transaction model, they develop a dynamic response to the author and take an active role in the construction of meaning, drawing on personal experiences, and critiquing the author’s message. These readers are not passive; rather, they actively interact with and respond to the author.

### Metacognitive strategies

Research has shown that text comprehension depends on directed cognitive effort to regulate text processing (Bazerman 1985;Pressley and Afflerbach 1995;van den Broek 1994;Wyatt et al. 1993). Skilled readers apply multiple strategies in a purposeful manner. These include setting reading goals, varying reading style according to the relevance of the text to reading goals, jumping forward and backward in the text to find information relevant to reading goals, making predictions about what the author will say, paraphrasing, explaining, and interpreting the text, and constructing summaries and conclusions. Skilled readers know multiple strategies and also know when to apply them (Garner 1987,1990).

Metacognitive strategies incorporate two subscales: analytic and pragmatic strategies (Taraban et al. 2004). The analytic subscale measures cognitively-based strategies related to processes like inference and evaluation (e.g., an item from the analytic subscale: *As I am reading, I evaluate the text to determine whether it contributes to my knowledge and understanding of the subject*). The pragmatic subscale measures action-based strategies that a student applies in order to remember or later find information (e.g., an item from the pragmatic subscale: *I make notes when reading in order to remember the information*). The analytic and pragmatic approaches relate to characteristically different cognitions related to comprehension.

### Information processing by non-native and native speakers

Research studies suggest that reading text in a non-native language affects information processing, specifically in reducing critical thinking. Vianty (2007) explored the difference in students’ use of metacognitive reading strategies in their native language, Bahasa Indonesia, and their second language, English. Participants completed the Metacognitive Reading Strategies Questionnaire (MRSQ – See Appendix A) in English and Bahasa Indonesia after completing a reading test in English and Bahasa Indonesia, respectively. The results showed that there was a difference in the types of metacognitive reading strategies employed while reading academic material in each of the two languages. Students used analytic strategies more in Bahasa Indonesia than English. Analytic strategies are associated with deep understanding of the material. Conversely, use of pragmatic strategies was higher when reading in English.^a^ Pragmatic strategies are used to highlight and underline important information in a text to remember it and to make it easier to find later. In that sense, pragmatic strategies assist in the basic selection, organization, and encoding of information. Razi (2010) administered the MRSQ to Turkish students before and after they participated in a study involving training in metacognitive strategy use. Of interest here were the pretest scores. Consistent with the data from Vianty (2007), Turkish students reading in English reported using pragmatic strategies more frequently than analytic strategies.^b^ Hayati and Shariatifar (2009) conducted a study with Iranian undergraduates who were learning English as a foreign language. After briefly training the students to apply knowledge mapping (i.e., a variant of concept mapping) or an underlining strategy, the researchers administered a reading comprehension test in English. The results showed significantly higher comprehension scores for students trained to use an underlining strategy (a pragmatic strategy in the MRSQ) compared to those trained to use knowledge mapping, suggesting that processing in a non-native language benefitted more from applying pragmatic strategies than analytic strategies. Together, these results indicate that information processing in a second language may be challenging and evokes basic and practical steps to comprehend and retain information more than the analytic and critical thinking processes readers would apply when reading in their native language.

Taraban (2011) examined metacognitive strategy use and epistemological beliefs in native-speaking engineering majors in the U.S. Consistent with the data of Vianty (2007) for processing in Bahasa Indonesia (the native language), analytic strategy use predominated over pragmatic strategy use. From freshman to senior years there was a small but significant increase in the use of metacognitive strategies. Taraban (2011) also showed that U.S. students express increasingly weaker transaction beliefs and increasingly stronger transmission beliefs, from freshman to senior years. This shift presumably reflects students’ need to focus on the factual nature of engineering and the direct transmission of information through reliable sources as they advance through training during their freshman to senior years.

## A cross-cultural study

The purpose of the present research was to apply the same constructs as those used by Taraban (2011) to a sample of Indian engineering students in order to examine information literacy from a cross-cultural perspective. The language of instruction in Indian universities is English; however, this is not typically the native language (the mother tongue) of the students. Indeed, India is home to several hundred languages. Hindi is the most widespread language. In the 2001 census, only 226,000 out of over one billion individuals (0.02% of total population) claimed Indian English as their native language. There were six hypotheses:

1. Based on previous research (Hayati and Shariatifar 2009;Razi 2010;Vianty 2007), we hypothesized that self-reports of pragmatic strategy use would predominate over reports of analytic strategy use for Indian students because these students used English in their academic work, which is not their native language.
2. For the same reason, we hypothesized that Indian students would report applying pragmatic strategies relatively more frequently than U.S. students.
3. Further, we hypothesized more growth in the reported use of both strategies by Indian students compared to U.S. students. Specifically, in countries like China, India, and the U.A.E, where there is a need to learn reading and writing for engineering in a non-native language (Bielenberg 2011), there is simply more room for growth and the possibility of larger effects over time.
  Hypotheses for the application of transmission and transaction beliefs follow on the logic of the previous hypotheses. By theory, transaction thinking is expressed and developed by engaging materials in an analytic and critical fashion.
4. To the extent that Indian readers are generally constrained to read academic materials at a pragmatic level, with English as their second language, we hypothesized that Indian students would affirm transmission beliefs more strongly than transaction beliefs, compared to a U.S. sample.
5. Conversely, U.S. students reading in their native language have more opportunities to apply transaction strategies; therefore, we predicted that U.S. students would exceed Indian students in their affirmation of transaction beliefs.
6. Based on the findings in Taraban (2011) that engineering students become more transmission-oriented from freshman to senior years, we further hypothesized that Indian students would replicate that pattern, due to comparable academic constraints in their development as engineers.

In Taraban (2011), the pattern of results was linked to the specific demands for information processing imposed on students through the curriculum and on students’ academic reading materials and activities. The Indian data in this study will be examined with an identical focus on students’ academic reading materials and activities.

## Method

### Participants

Three hundred thirteen students at an Indian Institute of Technology (IIT) participated in this study voluntarily and without compensation. These students represented a convenience sample.

In order to examine differences across freshman through senior levels, participants were classified based on completed credit hours: 0–50: freshman, 51–100: sophomore, 101–150: junior, 151 or more: senior. These ranges were confirmed by IIT faculty and are due to heavy course loads at IITs and concomitant long hours in lecture. The sample included 106 freshman, 86 sophomores, 50 juniors, and 71 seniors. The comparison sample (Taraban 2011) consisted of 410 engineering majors drawn from two large public research universities, Texas Tech University (*n* = 290) and the University of Wyoming (*n* = 120), which are in the southwest and west regions of the U.S., respectively. Both institutions have well-established engineering programs. The sample included 108 freshman, 93 sophomores, 102 juniors, and 107 seniors. There were 40 females in the Indian sample and 53 in the U.S. sample.

None of the participants was asked to report his or her native language, thus we can only report general statistics. Among Texas Tech engineering majors, 94 out of 2971 (3%) reported being non-resident aliens (Data were not available for the University of Wyoming). Thus, it appears that only a very small percentage of the U.S. sample was likely to be non-native English speakers. Hardly anyone in India is a native speaker of English. However, of the IIT applicants in 2011, about 60% were from English-medium schools, which are schools that use English as the medium of instruction where English is not the native language of the students. This suggests that a significant proportion of IIT students may have a working knowledge of English.

### Materials

The Reader Belief Inventory (RBI; Schraw 2000) and the Metacognitive Reading Strategies Questionnaire (MRSQ; Taraban et al. 2004) were two psychometric instruments used to gather the primary data. The two instruments provided different measures of information literacy. Within the psycholinguistic theories underlying these two instruments, beliefs and attitudes about text, as measured by the RBI, are conceptually distinct from the specific actions that individuals take in order to better comprehend text, as measured by the MRSQ. Further, measures of epistemological beliefs and metacognitive strategies apply to expository texts, which are the kinds of texts encountered in an engineering curriculum. Metacognitive strategies assist in constructing and using a coherent representation of information, including expository information, as evidenced, for example, in their applications in reading physics texts (Bazerman 1985). Similarly, Dai and Wang (2007) showed that epistemological beliefs are predictive of comprehension of expository texts. Therefore the two measures are appropriate for measuring text processing among engineering students. Finally, prior research has shown that the MRSQ and RBI subscales are relatively independent, and individuals can have any combination of high and low scores on the subscales.

The MRSQ included 20 items and RBI 11 items. The items for both scales are presented in Appendix A. Both scales used a 5-point Likert scale. The rating scale for the MRSQ, which measured frequency of strategy use, was specified as follows: *I use this strategy* 1-Never, 2- Rarely, 3-Sometimes, 4-Often, 5-Always. A sample item reads: *I make notes when reading in order to remember the information*. The rating scale for the RBI, which measured a person’s response to a statement, was specified as follows: *My response to this statement:* 1-Strongly Disagree, 2-Disagree, 3-Neutral, 4-Agree, 5-Strongly Agree. Prior to administering the questionnaire, the researcher met with a group of five graduate students in the Department of Humanities and Social Sciences at the Institute who evaluated the questionnaire for comprehensibility and appropriateness. The suggestions were to include an example question and response and to change GPA to CGPA in the questions. The latter term, used in India, represents a student’s cumulative grade-point average. In order to preserve paper at the IIT, the researcher was encouraged to reduce the number of questionnaire pages by making the format of the questionnaire more compact. In order to assure the equivalence of the U.S. and Indian formats, an independent experiment was subsequently conducted, comparing mean ratings for both formats. There were no significant differences in students’ ratings related to format for the RBI or MRSQ. Therefore, differences between U.S. and Indian students could not be attributed to the questionnaire formats. A detailed description of the experiment involving formats can be found in an additional file (see Additional file 1).

### Procedure

Participants were recruited through undergraduate engineering courses with the prior permission of the instructor. There was an attempt to target courses at every level from freshman through senior. The data were collected in a classroom setting. Students completed a consent form and were assured that their responses were confidential and would not affect their course grades. They then voluntarily completed English versions of the MRSQ and RBI. Four versions of each questionnaire were used, and the order of the MRSQ and RBI was counterbalanced across participants in order to eliminate spurious effects in the data due to a specific ordering of questions. Participants also responded to several demographic questions, questions about the amount of time spent reading and and completing homework activities, and an open-ended question about recent things they read. The instructions for the open-ended question were identical to those used with U.S. students, except that *Devdas* was used instead of *Moby Dick*, the former more familiar to Indian students. The instructions for the open-ended question were as follows:

List 3 recent and specific purposes or reasons for reading as part of your academic work. Avoid general responses like ‘to learn more.’ Acceptable responses are ‘I read the Sharat Chandra Chattopadhyay novella *Devdas* for Humanities class.’ ‘I read Ch. 3 in my Chemistry textbook in order to answer a problem set.’

The U.S. and Indian instructions directed the students to respond to the questionnaire items with respect to ‘reading materials for school’, and to the open-ended question with respect to their ‘academic work’. This was done to gather data specifically related to the academic culture at the respective institutions. Demographic questions, questions about time spent reading and completing homework, and open-ended questions always appeared at the end of the survey. The completed materials were returned to the researcher and participants were debriefed and dismissed.

### Confirmatory factor analysis and Cronbach’s alpha

Prior to analyzing the data, validity and reliability were assessed to gauge the appropriateness of scales for use with Indian engineering students. Validity is concerned with the extent to which the scales measured the mental constructs they were intended to measure. Reliability is concerned with whether the scales measured constructs consistently. The unidimensionality of scales, related to their validity, was assessed through the application of confirmatory factor analyses. The internal consistency of items in measuring a construct was estimated by computing Cronbach’s coefficient alpha.

Confirmatory factor analyses provide stringent tests of scale dimensionality because they require that the patterns of variable and factor relations be specified *a priori* in the form of hypotheses, which are then tested statistically, either confirming or disconfirming the hypothesized variable and factor relations. Confirmatory factor analyses are appropriate and recommended when verifying the applicability of a questionnaire to a new cohort of students (Brown 2006). To test the scale models, three goodness-of-fit measures were employed: the Standardized Root Mean Square Residual (SRMR; Brown 2006), the Comparative Fit Index (CFI; Bentler 1990), and the Root Mean Square Error of Approximation (RMSEA, Steiger 1990). These measures represent one index from each of three classes of fit measures: absolute, comparative, and parsimony, respectively. Acceptable values for these measures have been debated in the literature (e.g., Marsh et al. 2004), however, an acceptable fit of a model to observed data has been associated with the following values: SRMR values close to .08 or below; RMSEA values close to .06 or below; and CFI values of .95 or greater (Brown 2006;Hu and Bentler 1999). The MRSQ and RBI models were deemed to have a good fit to the data if these three criteria were met, although it is important to note that these goodness-of-fit measures are meant to function as “rules-of-thumb” rather than “golden rules” that specify absolute cutoff levels (Marsh et al. 2004). Others, for instance, have set an acceptable cutoff for the CFI at 0.80 (Meyers et al. 2006).

The previous results from the tests with U.S. students (Taraban 2011) are presented here for convenience. For the U.S. cohort, the following was found. For the MRSQ, the three critical indices for goodness-of-fit were a SRMR = .061, CFI = .854, and RMSEA = .056. For the RBI, the three critical outcomes were a SRMR = .047, CFI = .940, and RMSEA = .036. For the Indian cohort, for the MRSQ, the three critical indices for goodness-of-fit were a SRMR = .067, CFI = .810, and RMSEA = .055. For the RBI, the three critical outcomes were a SRMR = .060, CFI = .820, and RMSEA = .047. In three of four tests, the CFI measure was lower than the stringent .95 criterion, but within the .80 criterion. The three indices together suggested acceptable fits. Standardized factor loadings of items for nearly all the variables were statistically significant at *p* < .05 (one item in each model in the Indian tests > .05). The goodness-of-fit statistics and significant factor loadings confirmed that the MRSQ and RBI subscales were unidimensional and measured the intended mental constructs.

**Figure 1:**
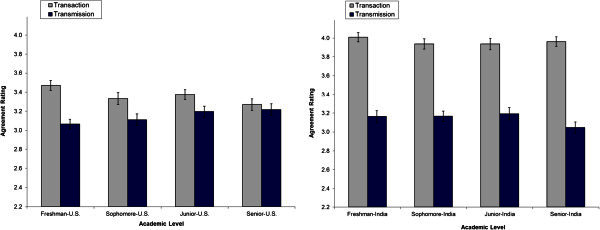
**Mean RBI ratings (*SEM*error bars) by level for U.S. and Indian students (U.S. data adapted from Taraban**^2011^**).**

In the data involving U.S. engineering students, Cronbach’s alphas were .80 and .81 for the analytic and pragmatic subscales, respectively, and .53 and .68 for the transaction and transmission subscales, respectively. In the current Indian data, Cronbach’s alphas were .69 and .76 for the analytic and pragmatic subscales, respectively, and .35 and .56 for the transaction and transmission subscales, respectively. The low alpha for the transaction scale (.35) was surprising and prompted additional investigation. Two of the five transaction questions had leptokurtic distributions, which tend to underestimate the true Cronbach alpha (Bay 1973;Sheng and Sheng 2012). Further, the Indian data were not normally distributed, based on Kolmogorov-Smirnov tests. Cronbach’s alpha is not recommended for establishing reliability with non-normal data (Sheng and Sheng 2012), thus the results need to be viewed with caution. Consistent with the high mean ratings by Indian students for transaction beliefs (see Figure 1), transaction question ratings are strongly negatively skewed, indicating a preponderance of high ratings.

### Questionnaire scoring and statistical methods

Each questionnaire was scored by taking the average of the ratings for each subscale (e.g., the analytic subscale). In the analyses of the MRSQ and RBI data, academic level (freshman through senior) based on completed credits is a between-subjects variable. Subscale ratings are within-subjects variables. Thus, the analysis of variance (ANOVA) used a mixed model design appropriate for these data. Non-parametric statistical methods were applied when there were doubts about the normal distribution of the data.

## Results

### Metacognitive strategy use

**Figure 2:**
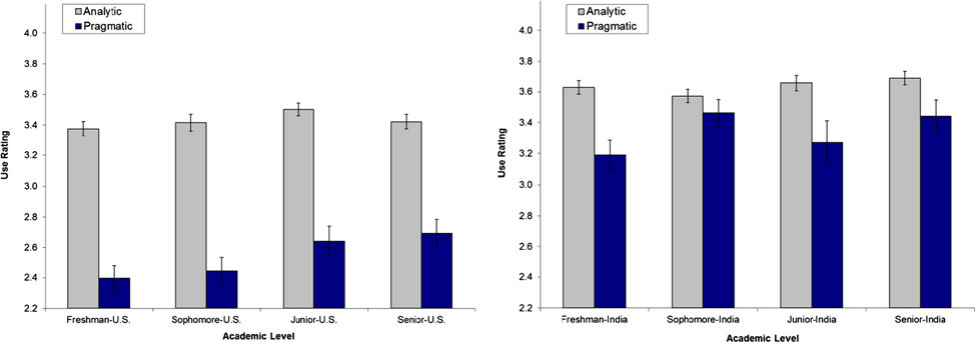
**Mean MRSQ ratings (*SEM*error bars) by level for U.S. and Indian students (U.S. data adapted from Taraban**^2011^**).**

Hypotheses were tested using ANOVAs to find out the main and interaction effects of interest. The results for the MRSQ are discussed first. Figure 2 shows the U.S. and the Indian data. *Hypothesis 1* that students’ reports of pragmatic strategy use would predominate over analytic strategy use for Indian students was not supported. Rather, students reported more frequent application of analytic strategies than pragmatic strategies [*F*(1, 312) = 30.49, *p* < .001]. *Hypothesis 2* that Indian students would report more frequent use of pragmatic strategies than U.S. students reported was supported [*F*(1, 721) = 128.36, *p* < .001]. *Hypothesis 3* of more growth in reports of use of both strategies by Indian students compared to U.S. students’ reports was evaluated through a 2 (Country: U.S., India) × 2 (Strategy: analytic, pragmatic) × 4 (Level: freshman, sophomore, junior, senior) ANOVA. There was a significant effect for Country [*F*(1, 715) = 146.62, *p* < .001] and for Level [*F*(3, 715) = 3.19, *p* = .023], but not for the Country X Level interaction [*F*(3, 715) = 0.85, *ns*], nor for the Country X Strategy X Level interaction [*F*(3, 715) = 1.54, *ns*], showing that strategy growth of Indian students did not exceed that of U.S. students. An examination of Figure 2 shows that strategy-use growth for U.S. and Indian students was due largely to students’ reports of growth in the use of pragmatic strategies. Additional analyses revealed that Indian students reported applying analytic strategies more frequently than U.S. students reported using these strategies [*F*(1, 721) = 35.52, *p* < .001]. The difference between the reported use of analytic and pragmatic strategies was smaller for Indian students (*M* = 0.30) than for U.S. students (*M* = 0.88) [*F*(1, 721) = 63.61, *p* < .001], suggesting that Indian students made frequent use of both types of strategies in contrast to U.S. students who showed a relative bias for analytic strategies over pragmatic strategies.

### Reader beliefs

Figure 1 shows the U.S. and the Indian data on RBI. A 2 (Country: U.S., India) × 2 (Belief: transaction, transmission) × 4 (Level: freshman, sophomore, junior, senior) ANOVA showed a main effect for Country [*F*(1, 715) = 103.55, *p* < .001], for Belief [*F*(1, 721) = 288.54, *p* < .001], and interaction effect for Country X Belief [*F*(1, 721) = 98.87, *p* < .001]. U.S. and Indian students did not differ in their ratings of transmission beliefs; however, Indian students affirmed transaction beliefs more strongly than U.S. students, as shown by the significant interaction. *Hypothesis 4* that Indian students would affirm transmission beliefs over transaction beliefs was not supported. *Hypothesis 5* that U.S. students’ affirmation of transaction strategies would exceed Indian students’ affirmation of these strategies was also not supported. *Hypothesis 6* that ratings of transaction and transmission beliefs would converge for Indian students, from freshman through senior years, as is evident in Figure 1 for U.S. students and reported in Taraban (2011), was not supported. Consistent with the finding in Taraban (2011), the Country × Belief × Level interaction was marginally significant [*F*(3, 715) = 2.34, *p* = .072].

### Academic times

The questionnaires that were administered to U.S. and Indian students included open-ended questions about their academic reading materials and activities. The responses to one set of questions asked students to report on times spent in a variety of academic activities (Note that the questions were intentionally *not* designed to probe mutually exclusive activities). U.S. students reported spending significantly more time doing homework and answering questions from the textbook, while Indian students reported spending significantly more time reading textbooks and other materials, like handouts, manuals, and novels (Table 1).

**Table 1:** **Mean times for academic activities for U.S. (*N*** **= 410) and Indian (*N*** **= 313) engineering students**

On average, each day I spend an average of ___ minutes	U.S.^a^	Indian	***p***-value^b^
Doing homework	122.48	44.14	**.001**
Reading textbooks for my classes	36.68	42.28	**.029**
Reading other printed materials for my classes (e.g., novels, handouts)	19.86	32.26	**.001**
Answering assigned questions from the textbook	68.13	22.44	**.001**
Writing papers for my classes	16.89	14.26	.079
Working on projects for my classes	31.81	32.28	.284

^a^U.S. data adapted from Taraban (2011). ^b^*p*-values (two-sided) based on Mann–Whitney *U* test for differences between mean ranks. Significant values are bolded.

The temporal data for each participant were correlated with each participant’s MRSQ and RBI scores. Examining Table 2, analytic strategy use was directly related with five of the six measures of time for Indian students, but only marginally for one measure of time for U.S. students. These positive correlations indicated that the more time students spent reading textbooks and other materials, like handbooks and novels, answering textbook questions, writing papers, and working on projects, the higher was their use of analytic comprehension strategies. The pattern was repeated for pragmatic strategies, with six out of six significant correlations for Indian students compared to three of six significant correlations and one marginal correlation for U.S. students. As already reported, Indian students reported significantly more use of analytic and pragmatic strategies than U.S. students, and the correlations linked those cognitive behaviors to academic work. A similar pattern was observed for transaction beliefs.

**Table 2:** **Spearman correlation coefficients for U.S. (*N*** **= 410) and Indian (*N*** **= 313) engineering students**

On average, each day I spend an average of ___ minutes	Analytic		Pragmatic		Transaction		Transmission	
	U.S.^a^	Indian	U.S.	Indian	U.S.	Indian	U.S.	Indian
Doing homework	.021	.073	^b^**.171**^**.001**^	**.176**^**.002**^	.043	.004	.041	**.149**^**.008**^
Reading textbooks for my classes	.036	**.159**^**.005**^	**.272**^**.001**^	**.168**^**.003**^	.057	**.124**^**.028**^	.070	.105^.063^
Reading other printed materials for my classes (e.g., novels, handouts)	.049	**.150**^**.008**^	**.161**^**.001**^	**.110**^**.050**^	.093^.059^	**.129**^**.023**^	-.003	.074
Answering assigned questions from the textbook	-.018	**.208**^**.001**^	.083^.094^	**.136**^**.016**^	-.051	.102^.071^	.064	.052
Writing papers for my classes	.091^.064^	**.130**^**.022**^	.063	**.157**^**.005**^	-.004	**.214**^**.001**^	-.049	**.136**^**.016**^
Working on projects for my classes	.065	**.133**^**.018**^	.079	**.159**^**.005**^	.014	.099^.080^	.048	-.016

^a^U.S. data adapted from Taraban (2011). ^b^*p*-values signifying the significance of the correlations are shown as superscripts. Two-tailed *p*-values < .05 are in bold; marginal *p*-values (.05 < *p* < .10) are shown but not bolded.

### Reading materials

U.S. and Indian students were asked to list three pieces that were read recently for academic work and the reasons for reading those materials. As shown in Table 3, Indian students listed fiction, manuals, reference books, magazines, and newspapers, significantly more often than U.S. students. U.S. students listed textbooks significantly more often than Indian students. Regarding purposes for reading, Indian students listed reading for exams, quizzes, for presentations or projects, and to learn about current events and to fulfill curiosity, significantly more often than U.S. students. U.S. students listed reading to answer homework sets and knowledge beyond the taught-materials more often than Indian students.

**Table 3:** **Mean percent of kinds of materials read by Indian (*N*** **= 313) and U.S. (*N*** **= 410) engineering students**

	Primary Sources			Secondary Sources							
	Non-Fiction	Journal/Article	Fiction	Textbook	Manual; Handbook; Guidebook; Reference	Website	Handout	Magazine; Newspaper	Student Class Notes	Unknown Source	SUM
Indian Students	5.33	4.44	13.10	52.72	6.22	3.98	3.55	4.55	5.33	0.78	100.00
U.S. Students^a^	6.33	1.04	3.92	61.69	1.94	1.56	2.56	1.27	3.46	15.93	100.00
MEAN	5.83	2.74	8.51	57.21	4.08	2.77	3.06	2.91	4.40	8.36	100.00
z-test^b^	0.41	n.a.^c^	4.36***	2.35*	2.82*	1.72	0.56	2.51*	1.00	n.a.	
**Mean Percent of Purposes for Reading**											
	**Answer Question Set/Homework**	**Exam/Quiz**	**Essay/Paper**	**Preview/Review (Before or after class)**	**Lab**	**Expand Knowledge (Beyond what was presented in class)**	**Applications of what was presented in class.**	**Pragmatic (to some academic end, e.g. raise GPA)**	**Fun/Pleasure Current Events/Curiosity**	**Presentation/Project**	**SUM**
Indian Students	7.66	33.30	0.44	13.54	2.44	8.44	0.22	6.88	18.42	8.66	100.00
U.S. Students	31.94	22.64	2.37	6.46	0	23.24	0.76	4.37	4.79	3.43	100.00
MEAN	19.80	27.97	1.41	10.00	1.22	15.84	0.49	5.63	11.61	6.05	100.00
z-test	7.80***	2.91**	n.a.	3.23**	n.a.	5.13***	n.a.	1.29	5.61***	2.89**	

^a^U.S. data adapted from Taraban (2011). ^b^The *z*-test is a difference test for two proportions, here comparing Indian and U.S. students columnwise. ^c^Because of statistical restrictions, tests were not conducted if either percent was based on a frequency less than 5. A *p*-value is considered significant if less than .05. Tests are two-tailed.

*** p < .001.

** p < .01.

*p < .05.

## Discussion

This study compared U.S. and Indian engineering students’ use of metacognitive strategies and their epistemic beliefs about text in academic settings. It is the first study of its kind to address the question of whether non-native speaking engineering students are inherently disadvantaged in information literacy and, by implication, in the global workplace. The hypothesis, motivated by findings in the published literature, was that Indian students, for whom English is not a native language, would be challenged by processing information in English. The results confirmed the hypothesis that Indian engineering students would apply pragmatic processing strategies more frequently than U.S. students. This result is consistent with the data in Vianty (2007), Razi (2010), and Hayati and Shariatifar (2009) concerning information processing in a non-native language. Pragmatic strategies are used to extract, organize, and retain basic information. It is plausible that Indian students rely more heavily on pragmatic strategies than U.S. students, in part, to compensate for reading outside their native language. The remaining hypotheses were not confirmed. The results showed that Indian students exceeded U.S. students in the use of analytic processing strategies. They also exceeded U.S. students in the affirmation of transaction beliefs. These latter effects implied that Indian students were more likely than U.S. students to actively process information through the application of metacognitive strategies, and to interpret, evaluate, critique, and respond to information, rather than simply accept information on the authority of the author.

Several questions asked students about the amount of time they spent engaged in academic activities. The significant positive correlations of these temporal data with the MRSQ and RBI subscales provided some validation for the scales themselves. Importantly, in relation to the pedagogical and cultural issues at hand, the correlations indicated that the demands placed on students through a curriculum, and students’ use of academic resources in the pursuit of academic goals, were positively associated with the application of metacognitive strategies and with critical and analytic thinking. For U.S. students, these associations were not nearly as reliable as they were for Indian students (as indicated by the magnitude of the correlations and strength of the *p*-values), implying that Indian students’ beliefs about information and their application of information-processing strategies were more tightly aligned with their engagement in typical academic activities.

Overall, Indian students read a wider variety of materials compared to U.S. students. These materials, like handbooks, reference books, and newspapers, may have naturally encouraged Indian students to read more reflectively, analytically, and critically. These differences in kinds of reading materials and purposes for reading may help to account for higher use of analytic and pragmatic strategies, and stronger affirmations of transaction beliefs, for Indian students compared to U.S. students. These data imply that analytic and pragmatic comprehension strategies and a transaction orientation to knowledge develop through extensive reading that goes beyond course textbooks and that involves additional goals besides those associated with completing textbook homework.

Academic norms and expectations -- the demands faculty and the curriculum place on students -- significantly determine the development of critical and analytic approaches to information. These curricular practices are not the same from one culture to the next (Bielenberg 2011), specifically, in the kinds of information that students process and the activities that faculty build around those materials. Overall the findings here and elsewhere provide support for ongoing initiatives to include more design projects, problem-based learning, cooperative education (co-op) experiences, and professional internships in engineering programs in order to continue to develop students’ abilities to analyze, interpret, critique, and respond personally to information, particularly in the context of ambiguity and the ill-defined problems that characterize professional practice (Felder and Brent 2004a, b;Marra and Palmer 2004).

In terms of the broad implications of these findings, the high achievements of Indian students in applying analytic strategies and affirming transaction beliefs beyond the levels of native speakers set a useful benchmark for all engineering undergraduates. Some instructional interventions have been successful in boosting metacognitive processing (Razi 2010) and critical thinking (Pavelich and Moore 1996). These interventions show that facilitating and supporting deliberate growth in information literacy is a viable possibility. Recommendations in *Educating the Engineer of 2020* (National Academy of Engineering 2004) include providing engineering majors with more breadth in the humanities. Including humanities courses across the freshman through senior years, particularly courses that include materials besides textbooks, may help students retain and develop a transactional and constructivist orientation to information and knowledge to a larger extent than is currently occurring. It may also assist them in more deeply appreciating the environmental, social, ethical, and political obligations that come with professional practice. There is a wealth of expository materials available to engineering students that probe humanistic issues, including sustainability, ethics, diversity, equality, gender, and social justice.

## Conclusions

The present study used a cross-sectional sample of IIT students who were working academically in a non-native language. A limitation to this study is that these Indian students represent a highly-select group in terms of motivation and ability. These students spoke more than one language, had mostly native-like English ability, and were avid readers. Therefore, the results reflect the performance of a sample with high-level English ability, albeit a non-native language. Future research should collect additional samples at other levels of ability and for other language groups. Non-native speakers of a language who do not have extensive exposure to that language may struggle to apply analytic strategies and transaction beliefs. Put differently, the results reported by Vianty (2007) should be replicated with other cohorts of engineering students, specifically Indian students with lower levels of English ability. Nonetheless, the findings from the study here show that one cannot assert generally that non-native speakers will be disadvantaged in information literacy in a non-native language.

There is wide acknowledgement of the value in training engineers as abstract thinkers, high-level problem solvers, and innovators (Gereffi et al. 2008) with good critical thinking skills (Bielenberg 2011) and an awareness of the importance of information literacy skills after graduation (Napp 2011). There is also recognition of the relevance and importance of developing intercultural communication and interpersonal professional training in order to prepare students for a global workplace (Mossbrucker et al. 2006). In order to determine what it means for engineering graduates to be’ globally competent” Klein-Gardner and Walker (2011) surveyed participants from academia and industry. The two most important competencies were the ability to communicate across cultures and the ability to appreciate other cultures. Up until now, hardly any research has been done to address the development of information literacy in engineering students. The present work begins to piece together comparisons of how students read and their beliefs about knowledge and information. Future research on information literacy will help to inform curriculum developers and instructors. The main goal is the development of an informed and capable workforce. It is difficult to conceive of future engineers who are not cognizant of professional and ethical issues and able to reflect on and respond to them responsibly and insightfully. This will depend on a lifelong ability to access and respond effectively to a variety of information sources. Finally, in light of results like those reported here, and additional confirmation, reports in the media that Indian students have weak English language skills may need to be more carefully qualified.

## Endnotes

^a^The two pragmatic strategies marked with an asterisk in Appendix A were used more frequently in Bahasa Indonesia than English.

^b^Excluding the two pragmatic strategies marked with an asterisk in Appendix A.

## Appendix A: Metacognitive Reading Strategies Questionnaire (MRSQ)

### Analytic reading strategies

1. As I am reading, I evaluate the text to determine whether it contributes to my knowledge/understanding of the subject.
2. After I have read a text, I anticipate how I will use the knowledge that I have gained from reading the text.
3. I try to draw on my knowledge of the topic to help me understand what I am reading.
4. While I am reading, I reconsider and revise my background knowledge about the topic, based on the text's content.
5. While I am reading, I reconsider and revise my prior questions about the topic, based on the text's content.
6. After I read the text, I consider other possible interpretations to determine whether I understood the text.
7. As I am reading, I distinguish between information that I already know and new information.
8. When information critical to my understanding of the text is not directly stated, I try to infer that information from the text.
9. I evaluate whether what I am reading is relevant to my reading goals.
10. I search out information relevant to my reading goals.
11. I anticipate information that will be presented later in the text.
12. While I am reading, I try to determine the meaning of unknown words that seem critical to the meaning of the text.
13. As I read along, I check whether I had anticipated the current information.
14. While reading, I exploit my personal strengths in order to better understand the text. If I am a good reader, I focus on the text; if I am good with figures and diagrams, I focus on that information.
15. While reading I visualize descriptions to better understand the text.
16. I note how hard or easy a text is to read.

### Pragmatic reading strategies

17. I make notes when reading in order to remember the information.
18. While reading, I underline and highlight important information in order to find it more easily later on.
19. While reading, I write questions and notes in the margin in order to better understand the text.
20. I try to underline when reading in order to remember the information.
*21. I read material more than once in order to remember the information.
*22. When I am having difficulty comprehending a text, I re-read the text.

## Reader Belief Inventory (RBI)

### Transaction statements

*23. I like the fact that two people can read the same book and disagree about what it means.
24. I often have strong emotional responses to what I read.
25. When I read, I like to imagine I am living through the experience too.
26. I enjoy interpreting what I read in a personal way.
27. Reading for pleasure is the best kind of reading.
28. I enjoy sharing the thoughts and reactions of characters in a book with others.

### Transmission statements

29. The main purpose of reading is to understand what the author says.
30. When I read, I try to carry away exactly what the author meant.
31. People should agree on what a book means.
32. I like books where you know exactly what the author means.
33. When I read, I focus on what the author says is important.
34. Most books mean exactly what they say.

*Notes*. Items marked with an asterisk were not used in the analyses and results, based on the recommendations generated in the confirmatory factor analyses in Taraban (2011). The rating scale for the MRSQ was as follows: *I use this strategy* 1-Never, 2- Rarely, 3-Sometimes, 4-Often, 5-Always. The rating scale for the RBI was as follows: *My response to this statement:* 1-Strongly Disagree, 2-Disagree, 3-Neutral, 4-Agree, 5-Strongly Agree. Four items in the RBI (Schraw 2000) that did not load (DNL) in excess of .40 on a factor in Taraban (2011) were not used in this study.

## Electronic supplementary material

Additional file 1: **Form test to compare U.S. and Indian questionnaire formats.** (DOC 48 KB)

## References

[CR1] Anand G (2011). The Wall Street Journal.

[CR2] Bay KS (1973). The effect of non-normality on the sampling distribution and standard error of reliability coefficient estimates under an analysis of variance model. Br J Math Stat Psychol.

[CR3] Bazerman C (1985). Physicists reading physics. Writ Comm.

[CR4] Bentler PM (1990). Comparative fit indexes in structural models. Psychol Bull.

[CR5] Bielenberg B: *Academic literacy: What is it? And why should design course faculty care?*. 2011. [*Proceedings of the American Society for Engineering Education Annual Conference & Exposition, Vancouver, BC, Canada*]

[CR6] Brown TA (2006). Confirmatory factor analysis for applied research.

[CR7] Dai DY, Wang X (2007). The role of need for cognition and reader beliefs in text comprehension and interest development. Contemp Educ Psychol.

[CR8] Farrell D, Laboissier M, Rosenfeld J, Sturze S, Umezawa F (2005). The emerging global labor market: Part II—the supply of offshore talent.

[CR9] Felder RM, Brent R (2004). The intellectual development of science and engineering students Part 1: Models and challenges. J Eng Educ.

[CR10] Felder RM, Brent R (2004). The intellectual development of science and engineering students Part 2: Teaching to promote growth. J Eng Educ.

[CR11] Garner R (1987). Metacognition and reading comprehension.

[CR12] Garner R (1990). When children and adults do not use learning strategies: Toward a theory of settings. Rev Educ Res.

[CR13] Gereffi G, Wadhwa V, Rissing B, Ong R (2008). Getting the numbers right: International engineering education in the United States, China, and India. J Eng Educ.

[CR14] Hayati AM, Shariatifar S (2009). Mapping strategies. J Coll Read Learn.

[CR15] Hu L, Bentler PM (1999). Cutoff criteria for fit indices in covariance structure analysis: Conventional criteria versus new alternatives. Structural Equation Modeling.

[CR16] (2012). India's engineering graduates cannot solve simple mathematical problems.

[CR17] Klein-Gardner SS, Walker A (2011). Defining global competence for engineering students. Proceedings of the American Society for Engineering Education Annual.

[CR18] MacAlpine B, Uddin M (2009). Integrating information literacy across the engineering design curriculum. Proceedings of the American Society for Engineering Education Annual.

[CR19] Marra R, Palmer B (2004). Encouraging intellectual growth: Senior college student profiles. J Adult Dev.

[CR20] Marsh HW, Hau K, Wen Z (2004). In search of golden rules: Comment on hypothesis-testing approaches to setting cutoff values for fit indices and dangers in overgeneralizing Hu and Bentler’s (1999) findings. Structural Equation Modeling.

[CR21] Mason L, Scirica F, Salvi L (2006). Effects of beliefs about meaning construction and task instructions on interpretation of narrative text. Contemp Educ Psychol.

[CR22] Meyers LS, Gamst G, Gurino AJ (2006). Applied multivariate statistics.

[CR23] Mossbrucker J, Petersen O, Scheibler S, Williams S, Wrate G: *Creating a ‘global algorithm’ for engineering education*. 2006. [*Proceedings of the American Society for Engineering Education Annual Conference & Exposition, Chicago, IL*]

[CR24] Napp JB: *Welcome to the real world: Showing the value of information literacy beyond the classroom*. 2011. [*Proceedings of the American Society for Engineering Education Annual Conference & Exposition, Vancouver, BC, Canada*]

[CR25] (2004). The Engineer of 2020.

[CR26] Pavelich MJ, Moore WS (1996). Measuring the effect of experiential education using the Perry Model. J Eng Educ.

[CR27] Pressley M, Afflerbach P (1995). Verbal protocols of reading: The nature of constructively responsive reading.

[CR28] Razi S: *Effects of a metacognitive reading program on the reading achievement and metacognitive strategies.*. Republic of Turkey: Dokuz Eylul University; 2010. [*Dissertation*]

[CR29] Schraw G (2000). Reader beliefs and meaning construction in narrative text. J Educ Psychol.

[CR30] Sheng Y, Sheng Z (2012). Is coefficient alpha robust to non-normal data?. Front Psychol.

[CR31] Shuman LJ, Besterfield-Sacre M, McGourty J (2005). The ABET ‘Professional Skills’ – Can they be taught? Can they be assessed?. J Eng Educ.

[CR32] Starkey A, Kissick B, Collins J, Oh J: *Faculty librarian partnerships for information fluency instruction: Planning and preliminary assessment. Proceedings of the American Society for Engineering Education Annual*. 2006. [*Proceedings of the American Society for Engineering Education Annual Conference & Exposition, Chicago, IL*]

[CR33] Steiger JH (1990). Structural model evaluation and modification: An interval estimation approach. Multivariate Behav Res.

[CR34] Taraban R (2011). Information fluency growth through engineering curricula: Analysis of students’ text-processing skills and beliefs. J Eng Educ.

[CR35] Taraban R, Kerr M, Rynearson K (2004). Analytic and pragmatic factors in college students’ metacognitive reading strategies. Read Psychol.

[CR36] van den Broek P, Gernsbacher M (1994). Comprehension and memory of narrative texts: Inferences and coherence. Handbook of psycholinguistics.

[CR37] Vianty M (2007). The comparison of students’ use of metacognitive reading strategies between reading in Bahasa Indonesia and in English. Int Educ J.

[CR38] Wyatt D, Pressley M, El-Dinary P, Stein S, Evans P, Brown R (1993). Comprehension strategies, worth and credibility monitoring, and evaluations: Cold and hot cognition when experts read professional articles that are important to them. Learn Indiv Differ.

