# Supplementary material for: Information literacy of U.S. and Indian engineering undergraduates
Source: Springerplus. 2013 May 27;2:244. doi: 10.1186/2193-1801-2-244 (PMC3679410; doi:10.1186/2193-1801-2-244)
Supplement: Supplementary file 1 — Additional file 1: Form test to compare U.S. and Indian questionnaire formats. (DOC 48 KB) [file 40064_2012_313_MOESM1_ESM.doc]

Supplemental Material

Information Literacy of U.S. and Indian Engineering Undergraduates

Roman Taraban

Department of Psychology

Texas Tech University

Lubbock, TX USA

[roman.taraban@ttu.edu](mailto:roman.taraban@ttu.edu)

Damodar Suar

Department of Humanities & Social Sciences

Indian Institute of Technology Kharagpur

West Bengal, India

[ds@hss.iitkgp.ernet.in](mailto:ds@hss.iitkgp.ernet.in)

Kristin Oliver

Department of Psychology*

Texas Tech University

Lubbock, TX USA

[kristin.oliver@ttu.edu](mailto:kristin.oliver@ttu.edu)

*currently in Department of Educational Leadership

Corresponding Author: Roman Taraban [roman.taraban@ttu.edu](mailto:roman.taraban@ttu.edu)

**Form Test to Compare U.S. and Indian Questionnaire Formats**

This post-hoc experiment was conducted to confirm that small differences between the U.S. and Indian questionnaire formats could not explain the significant differences in performance between the two groups. The primary differences were orally reading instructions to Indian participants as they followed along on the written instructions, including an example question (see text), and typing response possibilities (e.g., *I use this strategy* 1-Never, 2- Rarely, 3-Sometimes, 4-Often, 5-Always) at the top of each page (Indian format – short form) rather than after each question (U.S. format – long form). The latter change was implemented on the urging of Indian faculty to preserve paper.

Two different forms of each MRSQ-RBI questionnaire version were used when collecting data for the current study, resulting in four forms: long form of version A, short form of version A, long form of version B, and short form of version B. The order of questionnaire scales (MRSQ, RBI) was counterbalanced across versions A and B, but they were in the same order on the long form and short form.

**Participants**

One hundred and seventy undergraduate student volunteers were recruited from the Psychology Subject Pool at Texas Tech University and were compensated with extra credit for a course according to the operating policy of the Subject Pool. The mean age of participants was 19.72 (*SD* = 4.18) years. The majority of participants were females (69%). Classification was based on completed credit hours: 0-30, freshman; 31-60, sophomore; 61-90, junior; 91 and greater, senior. The majority of participants (51%) were freshman, 33% were sophomores, 11% were juniors, and 5% were seniors. The most frequent majors were psychology (14%), nursing (12%), biology (11%), exercise and sports science (9%), business (6%), and human development and family studies (6%). Eighty-six participants completed the short form and 84 participants completed the long form.

**Materials**

The materials were the MRSQ and RBI forms used to collect the data from the U.S. cohort, as reported in Taraban (2011) and the MRSQ and RBI forms used to collect the data in the current study. Except for the changes noted above, the forms were identical to those used in the original data collection.

**Procedure**

Participants completed the questionnaire in small groups of 1-4 participants. The researcher randomly selected the type of questionnaire given to participants in each session, and participants in the same session received the same questionnaire. For the long form, participants were instructed to read the instructions and complete the questionnaire. This was consistent with the original U.S. procedure. The procedure for the short form replicated that used in India. The researcher read the directions and examples for each section and asked participants to complete the first item of each section to ensure they filled out the questionnaire correctly. The reasoning for this was to be sure they understood what to write on the blank line next to each question and to highlight the difference between the scales in each section.

**Results**

A 2 (Form: long, short) X 2 (Strategy: analytic, pragmatic) ANOVA for the MRSQ showed a non-significant main effect of Form [*F* (1, 168) = 0.94, *p* = .334] and for Strategy [*F* (1, 168) = 1.01, *p* = .316]. Importantly, there was no significant interaction between Form and Strategy [*F* (1, 168) = 1.89, *p* = .171]. Strategy use on the two forms was similar for analytic (short: *M* = 3.30, *SD* = 0.80; long: *M* = 3.51, *SD* = 0.49) and pragmatic strategy use (short: *M* = 3.33, *SD* = 1.01; long *M* = 3.30, *SD* = 0.96). A 2 (Form: long, short) X 2 (Belief: transaction, transmission) ANOVA for the RBI showed no significant main effect of Form [*F* (1, 168) = 2.75, *p* = .099], a significant effect for Belief [*F* (1, 168) = 243.86, *p* = .001], and a non-significant interaction of the two factors [*F* (1, 168) = .00, *p* = .976]. Transaction beliefs (short form: *M* = 3.87, *SD* = 0.60; long form: *M* = 3.96, *SD* = 0.55) and transmission beliefs (short form: *M* = 2.91, *SD* = 0.51; long form: *M* = 3.00, *SD* = 0.52) were similar on the two forms.

**Conclusion**

The critical result here involves the non-significant interactions with form, which showed that analytic and pragmatic strategy responses, and transaction and transmission responses did not vary as a function of format. Thus we can reliably conclude that the differences reported in the main study were not due to variations in the form type.

**References**

Taraban, R (2011) Information fluency growth through engineering curricula: Analysis of students’ text-processing skills and beliefs. J Engineering Ed 100:397-416
